# An unusual case of cavitating pulmonary nodules: Lemierre’s syndrome with isolated involvement of the external jugular vein

**DOI:** 10.1259/bjrcr.20170093

**Published:** 2018-02-22

**Authors:** John Reicher, Sam Brooke, Dominic Arnold, Paul Counter, Alaa Abdelgalil

**Affiliations:** 1 North Cumbria University Hospitals NHS Trust, Carlisle, UK

## Abstract

A 65-year-old female presented with symptoms of tonsillitis and sepsis. Despite initial treatment with i.v. fluid and antibiotics, her condition deteriorated and she became hypoxaemic. CT pulmonary angiography showed no filling defects in the pulmonary arteries, but there were multiple cavitating lung nodules, initially thought to represent metastases. A subsequent contrast-enhanced CT of the neck and thorax demonstrated thrombosis of the left external jugular vein (EJV), leading to a revised diagnosis of Lemierre’s syndrome (*i.e.* septic embolization from jugular thrombophlebitis). Noteworthy aspects of the case include the initial misdiagnosis of the cavitating lung nodules by the reporting radiologist and the isolated involvement of the EJV—Lemierre’s syndrome usually involves the internal jugular vein. The case highlights the importance of septic emboli in the differential diagnosis of cavitating lung nodules, and of assessment of the EJV as well as internal jugular vein in the context of oropharyngeal infection.

## Clinical presentation

A 65-year-old female presented to the emergency department with a sore throat, neck pain, fevers and trismus. She was unable to swallow solids. Shortly before admission she had been prescribed oral phenoxymethylpenicillin (also known as penicillin V) for tonsillitis by her general practitioner in the community. She had no medical history of note, was not on any regular medications and had not undergone any relevant prior radiological investigations.

On examination, she was hypotensive (BP 95/61 mmHg) and tachypnoeic (respiratory rate 20/min). Oxygen saturations were 96% on room air. There was palpable neck swelling at level two on the left side. Flexible nasendoscopy showed left parapharyngeal swelling.

Blood tests revealed thrombocytopaenia (platelets 19 × 10^9^ l^–1^) and raised inflammatory markers (C-reactive protein 91 mg l^−1^, white cell count 23.1 × 10^9^ l^–1^, neutrophils 21.5 × 10^9^ l^–1^), urea (21.3 mmol l^−1^) and creatinine (94 µmol l^–1^).The working diagnosis was sepsis secondary to left-sided tonsillitis, and the patient was admitted under the otorhinolaryngology team for treatment with i.v. fluids, benzylpenicillin and metronidazole.

On Day 1 of admission, the patient complained of worsening shortness of breath. An arterial blood sample demonstrated hypoxaemia (pO_2_ 7.7 kPa) and D-dimers levels were elevated (2433 µmol ^–1^).

## Differential diagnosis

In addition to the clinical diagnosis of tonsillitis, the following were considered the most likely explanations for the acute hypoxaemia:

- Pulmonary embolism
- Pneumonia
- Acute respiratory distress syndrome (ARDS)
- Fluid overload related to intravenous fluid therapy

Pulmonary embolism was felt to be the most likely diagnosis, given the markedly elevated D-dimers levels; initial radiological investigations in the first 24 h of the admission included a chest radiograph and CT pulmonary angiogram to establish the cause of the hypoxaemia, and ultrasound of the neck to exclude a superficial collection or vascular complication associated with the tonsillitis.

## Imaging findings

### Chest X-ray (on the day of admission)

**Figure 1.**
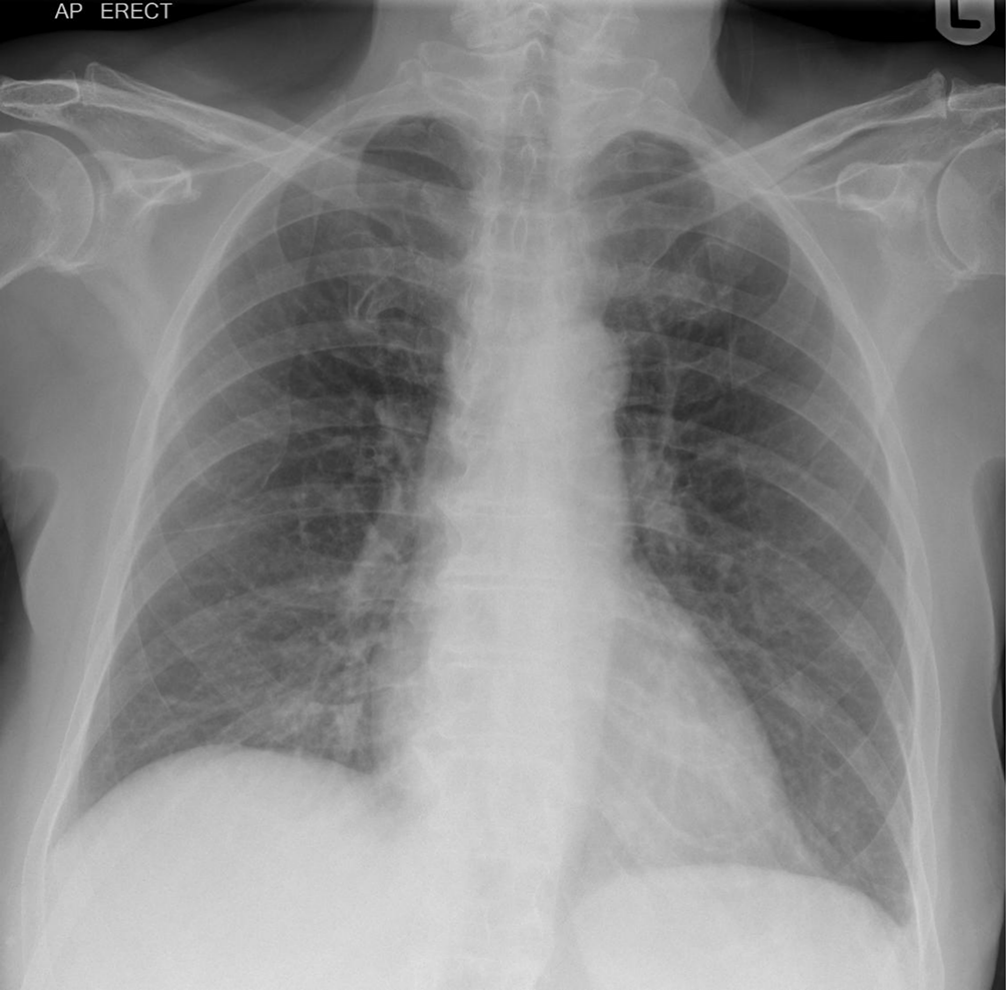
Anteroposterior frontal chest radiograph, showing no significant abnormality.

AP frontal chest radiograph showed no significant abnormality (Figure 1).

### CT pulmonary angiogram (1 day after admission)

**Figure 2.**
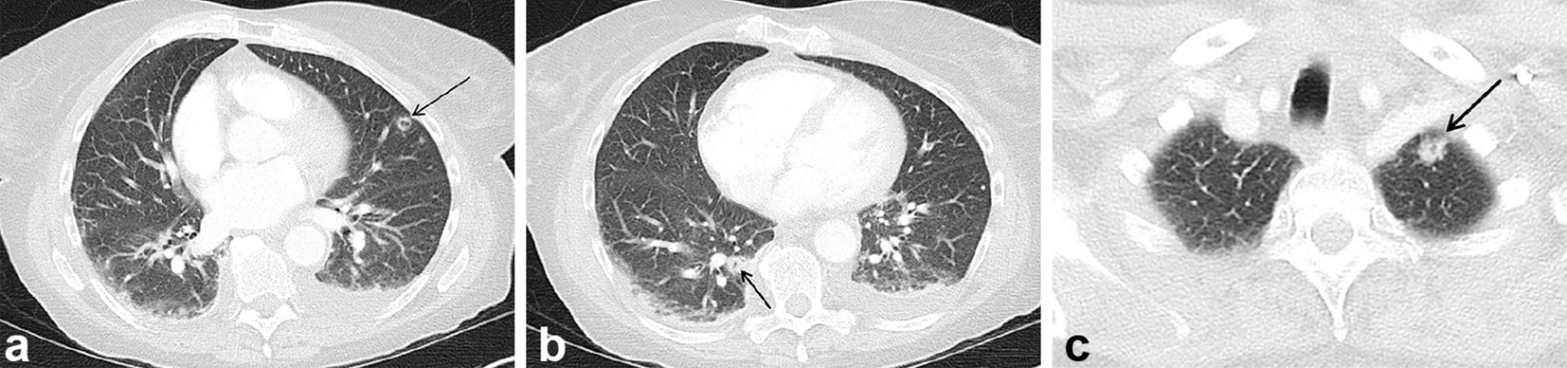
Axial images from the CT pulmonary angiogram on lung window, demonstrating multiple cavitating lung nodules (black arrows).

**Figure 3.**
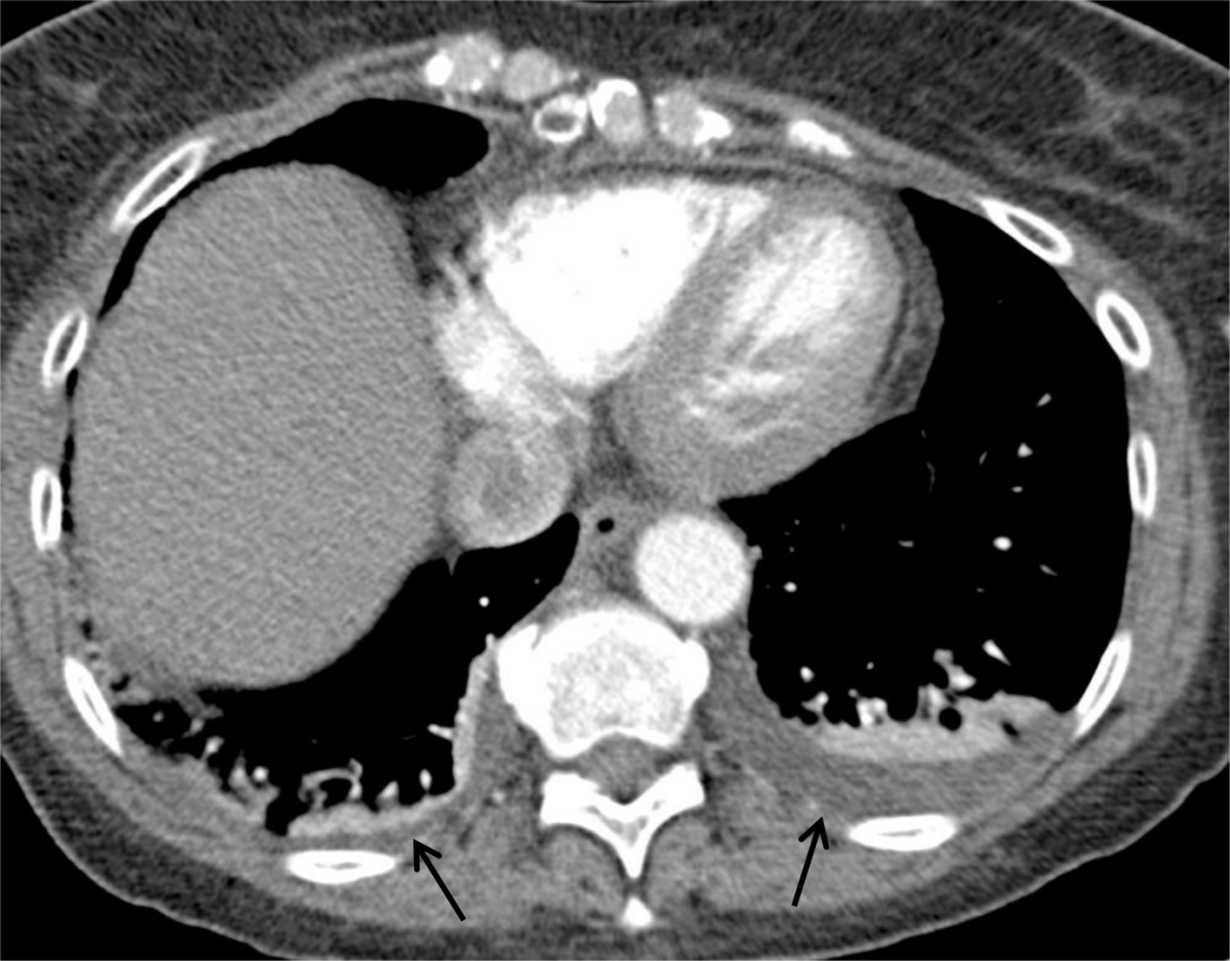
Axial image from the CT pulmonary angiogram on soft tissue window. Black arrows point to bilateral pleural effusions, worse on the left side, with some collapse in the adjacent lower lobes.

CT pulmonary angiogram demonstrated good opacification of the pulmonary arteries with no filling defects. There were multiple, ill-defined lung nodules measuring up to 11 mm, three of which showed central cavitation, with no specific zonal predilection. Wall thickness of the cavities was variable, from 1 mm up to 5 mm (Figure 2). Small bilateral pleural effusions (more prominent on the left side) were demonstrated (Figure 3).

The differential diagnosis of multiple cavitating lung lesions, which is explored in greater detail below, includes metastatic malignancy, septic emboli and many other causes.^1, 2^ The requesting information for this scan included unilateral neck swelling but did not emphasize the clinical features of sepsis or tonsillitis, and therefore the reporting radiologist considered the likeliest explanation of the CT findings to be metastatic malignancy from a possible head and neck squamous cell carcinoma.

### Ultrasound neck (1 day after admission)

**Figure 4.**
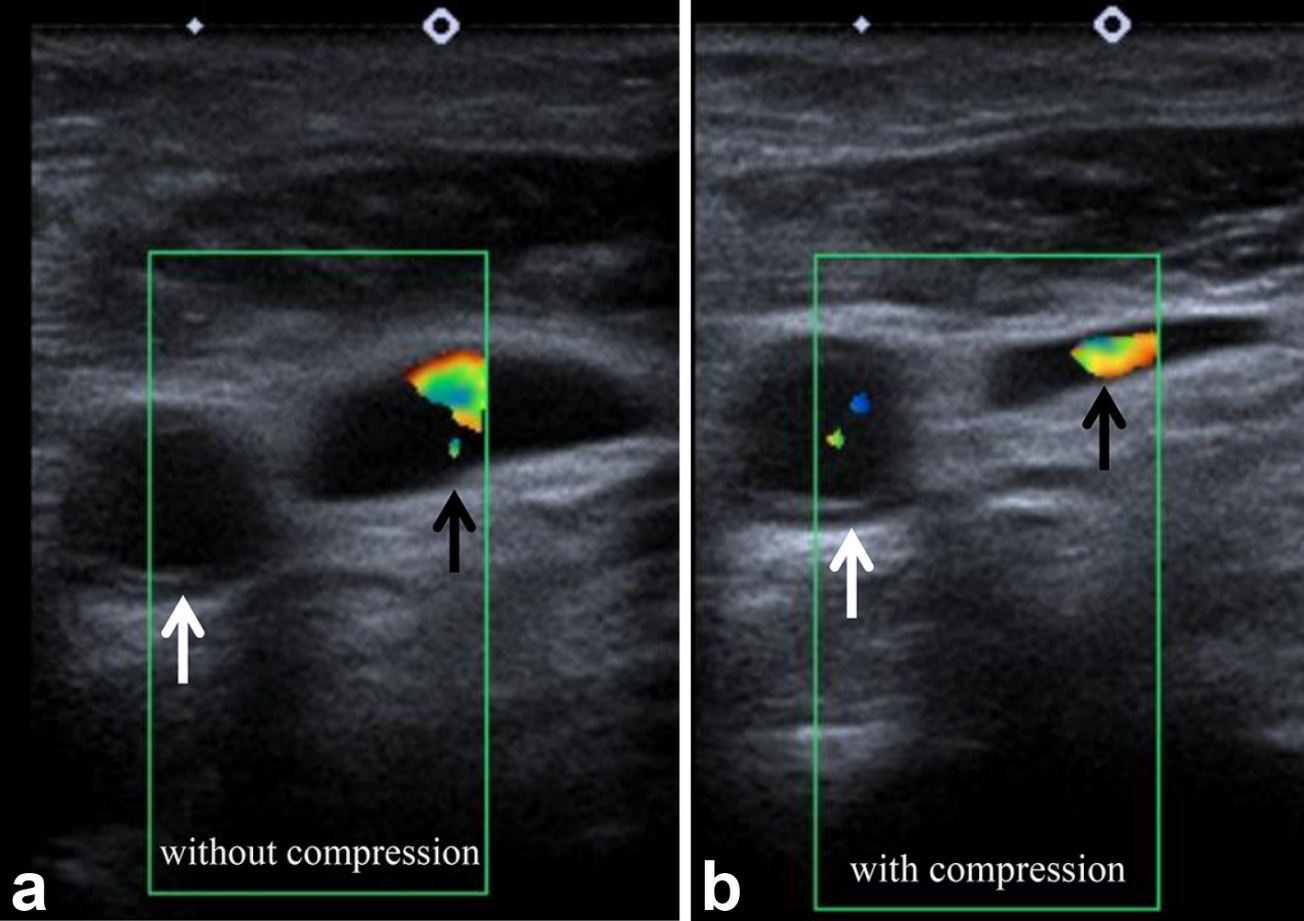
Ultrasound images of the left side of the neck, demonstrating some Doppler flow in the internal jugular vein (black arrows) and common carotid artery (white arrows), with a reduction in calibre of the internal jugular vein with probe compression.

Ultrasound of the neck showed no superficial collection or lymphadenopathy and normal appearances of the internal jugular veins (IJVs) and common carotid arteries bilaterally. In particular, compressibility of and Doppler flow within the left IJV were demonstrated (Figure 4). The external jugular veins (EJVs) were not assessed.

### CT neck/chest with IV contrast (5 days after admission)

Antibiotic therapy was continued, with no significant change in the patient’s clinical condition over the next 4 days. In response to the report for the CT pulmonary angiogram, which raised the possibility of cavitating lung metastases from a possible head and neck primary, the patient had a further CT scan of the neck and chest with i.v. contrast (in the venous phase), principally to assess for a primary malignancy.

**Figure 5.**
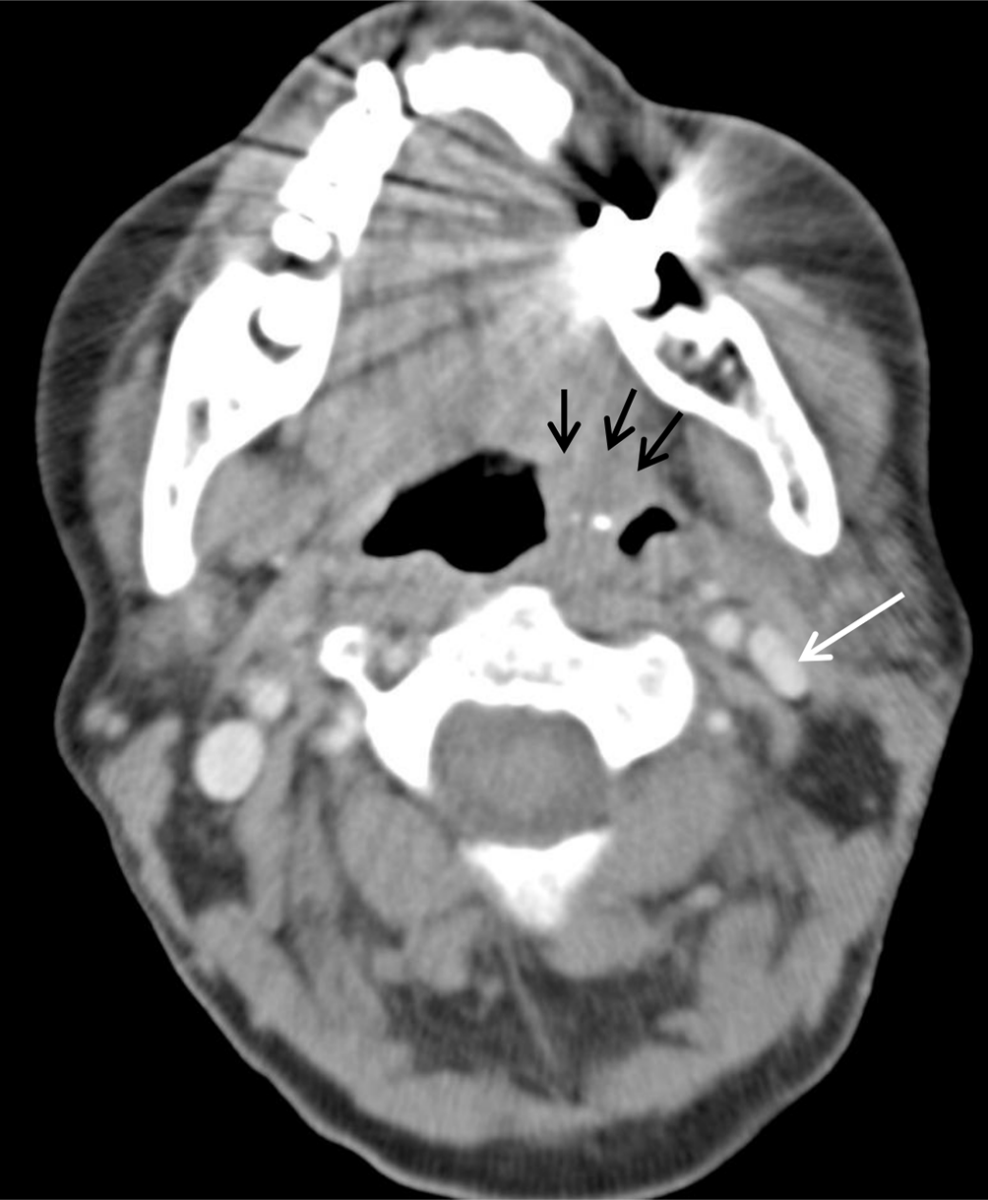
Axial image from the CT neck/thorax at the level of the oropharynx: swelling and a few flecks of calcification in the left palatine tonsil (black arrows). Note the normal enhancement of the internal jugular vein (white arrow).

**Figure 6.**
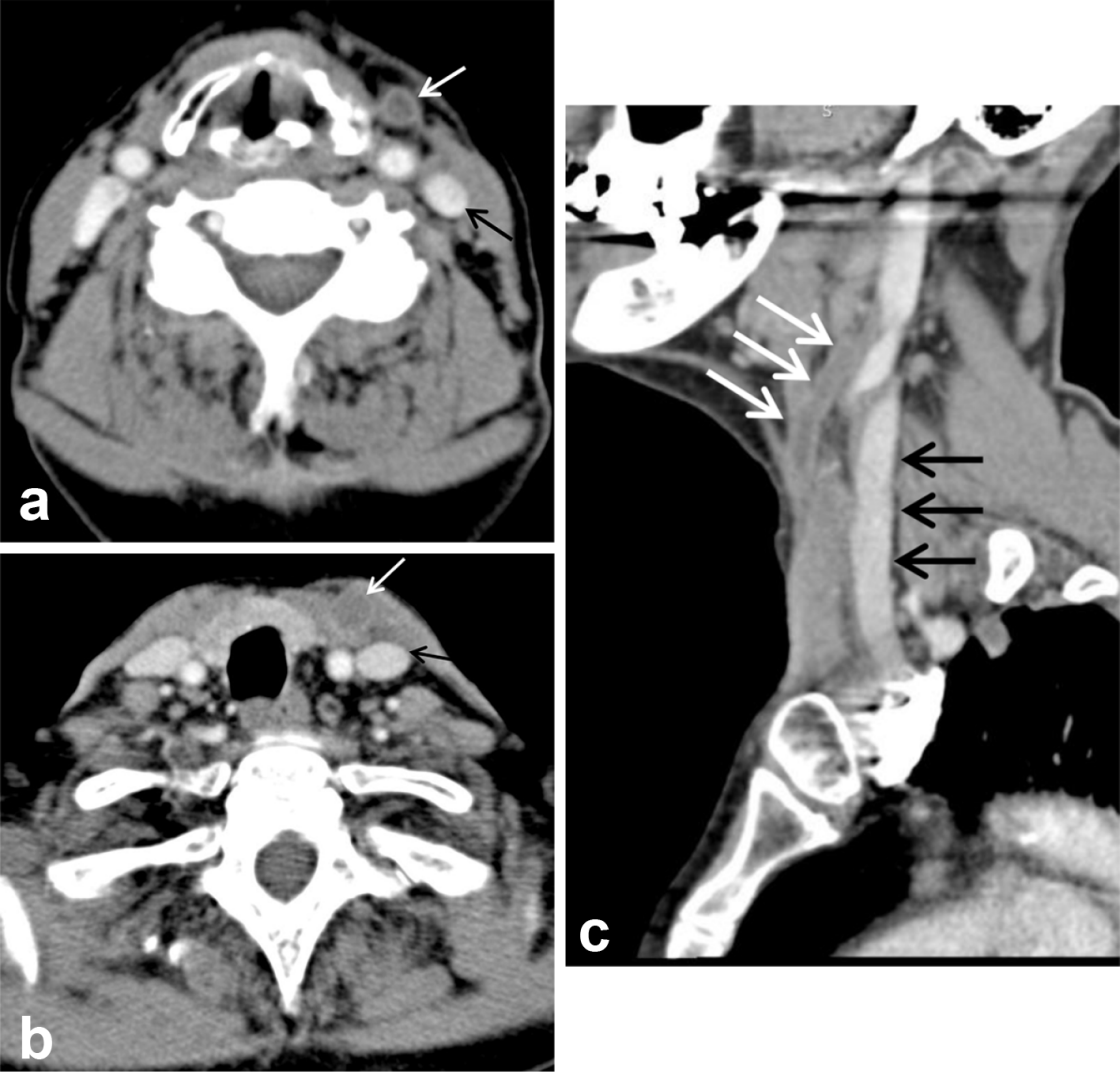
(Axial images through the neck from the CT neck/thorax) and (c) (sagittal image of the neck from the CT neck/thorax): white arrows point to the left external jugular vein, expanded compared with the contralateral side, with non-enhancing central thrombus. Black arrows point to the patent left internal jugular vein.

**Figure 7.**
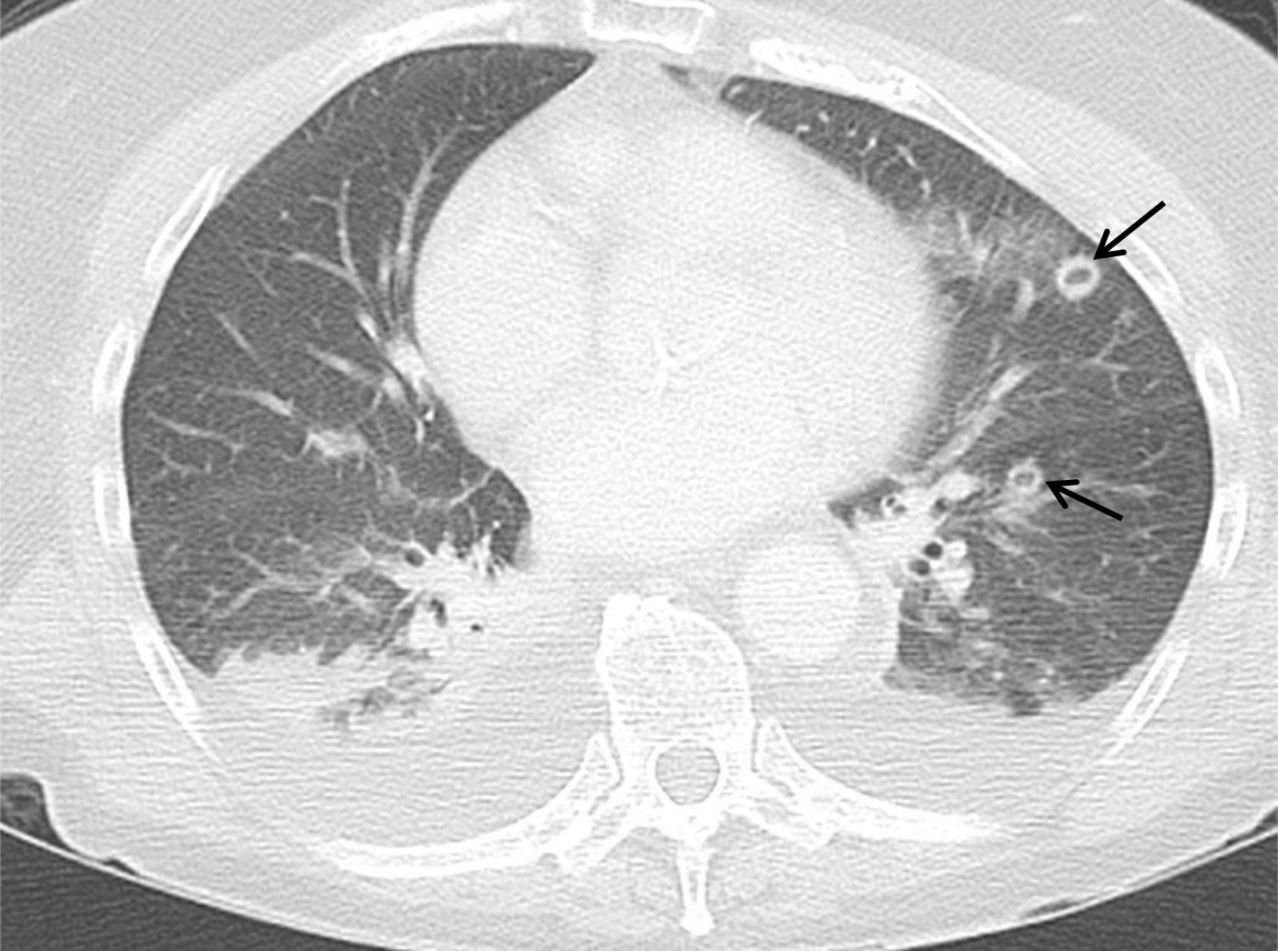
Axial image from the CT neck/thorax on lung window, at the same level as Figure 2. Cavitating lung nodules (black arrows) have increased in size and number compared with the initial CT pulmonary angiogram.

**Figure 8.**
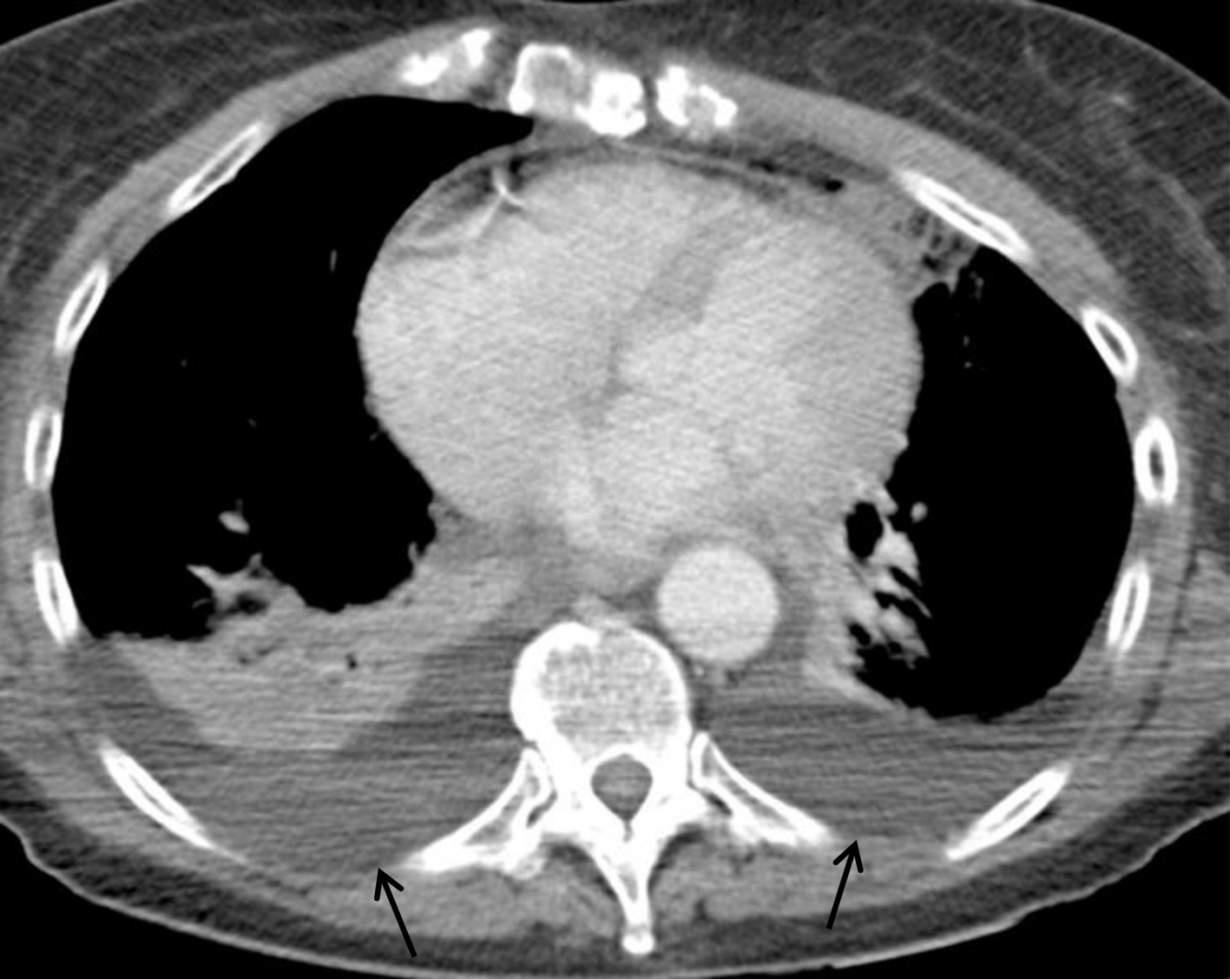
Axial image from the CT neck/thorax on soft tissue window, which demonstrates enlarging bilateral pleural effusions (black arrows).

This scan demonstrated swelling of the left palatine tonsil, which contained multiple flecks of calcification, in keeping with the clinical diagnosis of acute tonsillitis. The IJV demonstrated normal opacification (Figures 5 and 6). The EJV was markedly expanded by low attenuation thrombus (Figure 6). The cavitating lung nodules were now slightly larger (measuring up to 12 mm) and more numerous compared to the CT pulmonary angiogram (Figure 7 *vs* Figure 2) and the pleural effusions had increased in volume (Figure 8).These findings led to a revised diagnosis of septic pulmonary emboli from left EJV thrombophlebitis, secondary to ipsilateral tonsillitis (*i.e.* Lemierre’s syndrome, discussed in detail below).

**Figure 9.**
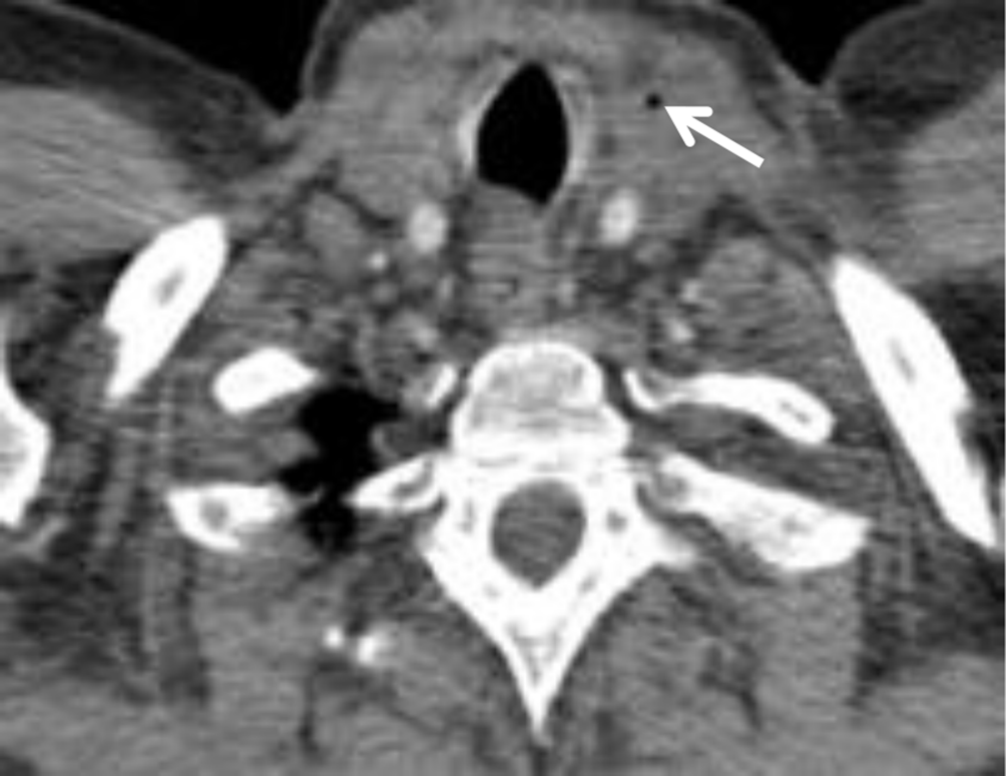
Axial image of the lower neck from the CT pulmonary angiogram: in retrospect, there is a small locule of gas in the region of the left external jugular vein. This is the only direct sign of external jugular vein thrombophlebitis on this scan given the phase of contrast.

In retrospect, although the systemic veins were not opacified on the initial CT pulmonary angiogram, owing to the phase of the scan, a small locule of gas was present in the left paratracheal soft tissue (Figure 9). This corresponds to the position of the thrombosed EJV on the subsequent contrast-enhanced neck CT (Figure 6). In hindsight this locule of gas is the only direct sign of septic EJV thrombus on the initial CT pulmonary angiogram.

## Outcome, follow-up and discussion

The patient was commenced on low molecular weight heparin, which was subsequently switched to an oral anticoagulant. No causative bacterium was isolated, despite multiple blood cultures and tonsillar swabs, but the patient improved on intravenous broad spectrum antibiotics and subsequently completed a 4-week course of oral co-amoxiclav. She was discharged from hospital on the sixth day of the admission, once tolerating oral medication and no longer dependent on supplemental oxygen.

**Figure 10.**
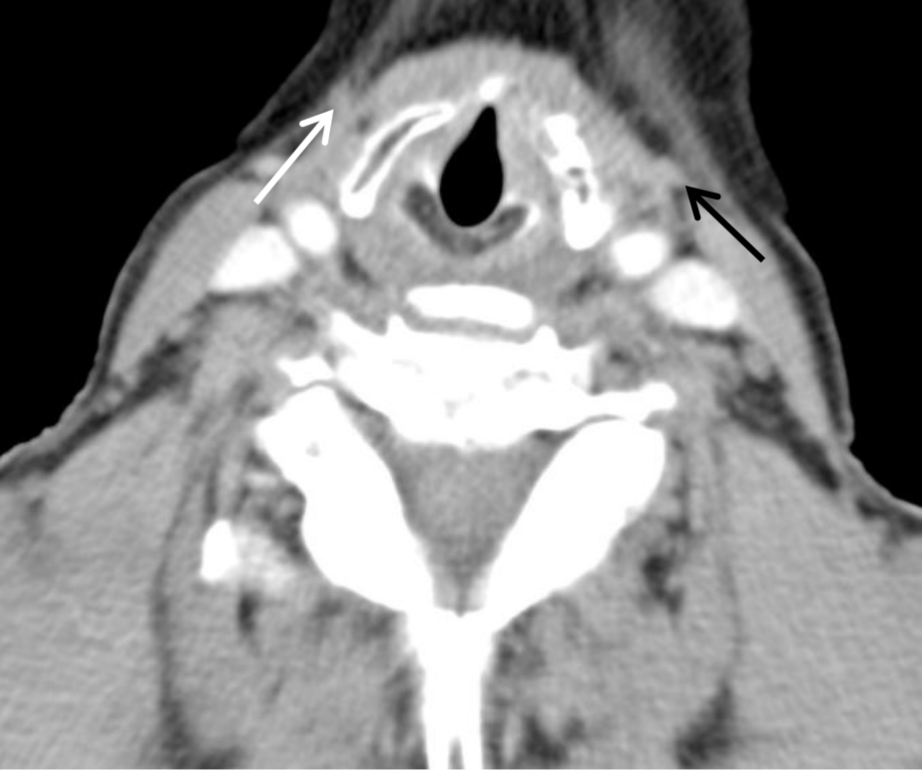
Axial image of the neck on the follow-up CT neck/chest demonstrating the left external jugular vein (black arrow) has returned to normal calibre and now enhances to the same degree as the contralateral external jugular vein (white arrow).

She remained under fortnightly outpatient review and continued to improve clinically. A follow-up contrast-enhanced CT neck and chest was performed at 30 days after the date of admission. This showed resolution of the left EJV thrombophlebitis (Figure 10), and a reduction in the size and number of the lung nodules. The pleural effusion had also reduced in size. The patient’s symptoms and blood tests (including the inflammatory markers and thrombocytopaenia) resolved. Antibiotics and anticoagulation were discontinued.

### Differential diagnosis of multiple cavitating lung nodules

A wide range of infective, inflammatory and ischaemic pathologies can cause pulmonary cavitation, which is usually a result of parenchymal necrosis.^1^ The differential diagnosis of multiple cavitating lung nodules includes metastatic malignancy, septic emboli, mycobacterial infection, autoimmune causes (*e.g.* systemic vasculitis) and pneumoconiosis.^2^

Within the wide spectrum of possible radiological appearances of septic pulmonary emboli, the classic picture is multiple, peripherally located, ill-defined nodules with cavitation, peripheral enhancement and lower zone predilection.^3, 4^ While these characteristics may help distinguish septic emboli from other causes—for example, mycobacterial disease, which typically displays an apical predilection—there is considerable overlap in the radiological appearances of the above differential diagnoses.^2^ While there have been studies demonstrating that wall thickness and enhancement characteristics of *solitary* pulmonary cavities can aid the distinction between benign and malignant disease,^5, 6^ it is unclear whether such rules can be reliably applied in the presence of *multiple *cavities.

Given these potential difficulties in reaching a diagnosis on radiological grounds alone, additional investigations such as percutaneous biopsy may be helpful, although this may not be feasible,^7^ especially in the context of small lesions in an acutely unwell patient. The clinical presentation, therefore, is often the most useful discriminator. In the above case, the initial CT pulmonary angiogram demonstrated only three cavitating nodules of variable wall thickness, with no zonal predilection. Meaningful analysis of nodule enhancement was precluded by the scan protocol. While these are relatively non-specific appearances, if the radiologist reporting the scan had fully appreciated the clinical background of tonsillitis and systemic sepsis, then septic emboli, rather than metastases, would have been the main differential diagnosis from the start.

### Lemierre’s syndrome

Lemierre’s syndrome, otherwise known as post-anginal sepsis, consists of metastatic infection from jugular thrombophlebitis. It is a rare complication of oropharyngeal infection with an estimated incidence in the general population of one case per million per year.^8^ Initially described in 1900, and later named after Dr André Lemierre following a case series he published in 1936,^9^ it usually occurs in the context of tonsillar or peri-tonsillar infection in young patients and classically involves the IJV.^10^ The most common causative organisms are anaerobic gram-negative bacilli from the genus Fusobacterium (especially *Fusobacterium necrophorum*).^11^ Less commonly, a gram-positive organism such as Streptococcus is implicated.^12^ The typical site of septic embolization is the lung, although metastatic septic arthritis, osteomyelitis and meningitis have been described.^13, 14^ Thrombocytopaenia, which resolves in parallel with clinical improvement, as observed in this case, is a characteristic feature.^12^

Lemierre’s syndrome is thought to involve the IJV in most cases as the venous drainage of the oropharyngeal mucosa and tonsils is typically to the IJV.^15^ In some patients, venous connections between the oropharyngeal veins and the EJV have been observed;^16^ if present, these allow direct propagation of thrombophlebitis into the EJV in the presence of oropharyngeal infection. These variant venous connections are thought to be responsible for the five previously published cases of isolated EJV involvement.^15,17–20^

The mainstay of treatment is antibiotic therapy, with gram-positive and anaerobic cover in the absence of an isolated organism. Although the subject is controversial, and definitive evidence is lacking, some experts advocate concurrent anticoagulation.^21^ In the pre-antibiotic era, the condition was almost invariably fatal: 18 out of the 20 patients in Lemierre’s original case series did not survive.^9^ In those days, IJV excision or ligation was the only viable treatment.^22^ However, today most patients given prompt antibiotic therapy make a good recovery.^23^

## Conclusions

The above case raises a number of important lessons for the general radiologist. The possibility of septic embolization from isolated EJV thrombophlebitis serves as a reminder to examine closely the EJVs as well as the IJVs, particularly in the context of oropharyngeal infection. In this case, the failure to assess the EJVs on ultrasound delayed the diagnosis. On the original CT pulmonary angiogram, in which the neck vessels were not opacified, the only sign of EJV thrombosis was a single small locule of gas—this highlights the value of contrast-enhanced CT of the neck in complicated oropharyngeal infection. Given the initial misdiagnosis of the cavitating pulmonary nodules as metastatic malignancy, the case also supports the general principle that radiologists should exercise caution in making a presumptive diagnosis of malignancy in a patient presenting with acute infective symptoms. Finally, adequate clinical information is essential for accurate interpretation of non-specific radiological findings. Definitive diagnosis of cavitating pulmonary nodules on the basis of radiological features alone may be challenging, particularly as the differential diagnoses have variable and overlapping appearances. However, in the context of tonsillitis and sepsis, the radiologist should suspect metastatic infection as the likeliest cause.

## Learning Points

1. Alternative diagnoses can be missed on CT pulmonary angiography due to the lack of systemic vascular enhancement.
2. Be wary of making a new diagnosis of malignancy in a patient acutely unwell with clinical features of sepsis. Consider whether an infective aetiology could explain the radiological findings.
3. Septic embolization is an important differential diagnosis for cavitating lung nodules, particularly in the context of ENT infection and/or thrombophlebitis.
4. Vascular imaging of the neck (*e.g.* contrast-enhanced CT) is indicated if pulmonary cavities are demonstrated in a patient with acute oropharyngeal infection.
5. Although classically Lemierre’s syndrome involves the IJV, isolated involvement of the EJV (as seen in this case) has been described and therefore the EJV should also be closely inspected in the context of oropharyngeal infection.

## Normal ranges

The following normal ranges are provided by the local laboratory where the above blood tests were carried out.

Platelets: 250–400 × 10^9^ l^–1^

C-reactive protein:<5 mg l^−1^

White blood cell count: 4−11 × 10^9^ l^–1^

Neutrophils: 1.8–7.5 × 10^9^ l^–1^

Urea: 2.5–7.8 mmol l^−1^

Creatinine: 49–90 µmol l^–1^

Arterial pO_2_: 12.0–14.6 kPa

D-dimers: <650 µg l^−1^
